# Secure ECDSA SRAM-PUF Based on Universal Single/Double Scalar Multiplication Architecture

**DOI:** 10.3390/mi15040552

**Published:** 2024-04-21

**Authors:** Jingqi Zhang, Zhiming Chen, Xiang He, Kuanhao Liu, Yue Hao, Mingzhi Ma, Weijiang Wang, Hua Dang, Xiangnan Li

**Affiliations:** 1 School of Integrated Circuits and Electronics, Beijing Institute of Technology, Beijing 100081, China; zhangjq@bit.edu.cn (J.Z.);; 2 UNISOC (Shanghai) Technology Co., Ltd., Shanghai 201203, China; 3 BIT Chongqing Institute of Microelectronics and Microsystems, Chongqing 401332, China; 4 School of Information and Electronics, Beijing Institute of Technology, Beijing 100081, China

**Keywords:** SRAM-PUF, elliptic curve digital signature algorithm, elliptic curve scalar multiplication, elliptic curve double scalar multiplication

## Abstract

Physically unclonable functions (PUFs) are crucial for enhancing cybersecurity by providing unique, intrinsic identifiers for electronic devices, thus ensuring their authenticity and preventing unauthorized cloning. The SRAM-PUF, characterized by its simple structure and ease of implementation in various scenarios, has gained widespread usage. The soft-decision Reed–Muller (RM) code, an error correction code, is commonly employed in these designs. This paper introduces the design of an RM code soft-decision attack algorithm to reveal its potential security risks. To address this problem, we propose a soft-decision SRAM-PUF structure based on the elliptic curve digital signature algorithm (ECDSA). To improve the processing speed of the proposed secure SRAM-PUF, we propose a custom ECDSA scheme. Further, we also propose a universal architecture for the critical operations in ECDSA, elliptic curve scalar multiplication (ECSM), and elliptic curve double scalar multiplication (ECDSM) based on the differential addition chain (DAC). For ECSMs, iterations can be performed directly; for ECDSMs, a two-dimensional DAC is constructed through precomputation, followed by iterations. Moreover, due to the high similarity of ECSM and ECDSM data paths, this universal architecture saves hardware resources. Our design is implemented on a field-programmable gate array (FPGA) and an application-specific integrated circuit (ASIC) using a Xilinx Virtex-7 and an TSMC 40 nm process. Compared to existing research, our design exhibits a lower bit error rate ($2.7\times 10^{-10}$) and better area–time performance (3902 slices, 6.615 μs ECDSM latency).

## 1. Introduction

### 1.1. Background

With the rapid expansion of the Internet of Things (IoTs), more devices require internet connectivity. Ensuring the security of integrated circuits in these devices is crucial to protect them from potential attacks [1,2]. The physical unclonable function (PUF) is an essential security technique for integrated circuits; it generates a unique electronic signature for each chip by exploiting chip characteristics caused by process variations [3]. PUF functions produce an output responding to a given challenge, forming a challenge–response pair (CRP). PUFs are classified into strong or weak based on their number of CRPs. Strong PUFs have numerous CRPs, while weak PUFs have only a few, which are usually used for key generation through direct response or hash transformation. Weak PUFs often require error correction circuits for reliability.

Among weak PUF circuits, the static random access memory (SRAM)-PUF is widely used due to its superior error correction and ease of implementation [4]. In SRAM-PUF, the initial value of SRAM serves as the PUF response upon power-on, ensuring no sensitive information is stored when powered off [5]. This method offers flexibility in key generation and high entropy due to the inherent randomness in physics.

Although SRAM-PUF is an “electronic fingerprint”, it is sensitive to noise and fabrication processes, leading to bit errors in the initial power-on value. To mitigate this, people adopt fuzzy extractor algorithms and categorize them into either hard-decision or soft-decision. Soft-decision algorithms are more robust in noisy conditions and utilize multiple sampling, which is often achieved by repeatedly powering SRAMs up and down. Reed–Muller (RM) code, a common soft-decision error correction code, is typically used, with pre-computed error probabilities $P_{error}$ stored in read-only memory (ROM) during registration. The soft-decision SRAM-PUF based on RM code is presented in Appendix A.

### 1.2. Related Works

As interest in PUFs grows, more and more potential attacks against PUFs have been proposed. Protocol attacks on PUFs are outlined in [6]. Additionally, refs. [7,8] detail various protocol attack strategies, including accessing the PUF temporarily, reusing previous PUF sessions, establishing stealth channels for malicious activities, and exploiting error correction schemes for information leakage. Furthermore, refs. [9,10] suggest a silicon attack method involving invasive techniques to manipulate or explore all possible PUF values or alter chip PUF values. Despite SRAM-PUF’s ability to resist some attacks, ensuring absolute security solely through initial values remains a challenge. For instance, in practical settings, the CPU’s connection to SRAM enables access to SRAM data through software programs, potentially exposing its initial value, as mentioned in [11]. Even if SRAM is not directly linked to the CPU, invasive methods like scanning electron microscopes or thermal laser stimulation can also be deployed to measure initial values [12].

Due to the risks associated with initial SRAM value leakage, this paper proposes a modified SRAM-PUF algorithm to address security concerns and enhance SRAM-PUF technology with the help of elliptic curve cryptography (ECC). The elliptic curve digital signature algorithm (ECDSA) can ensure confidentiality and is applicable for maintaining data integrity and authenticity [13]. Therefore, the initial SRAM value leakage problem can be solved. In ECDSA, curves are defined over prime fields and binary fields [14]. Binary fields offer advantages in modular operations due to their carry-free property, making ECC over binary fields more suitable for hardware implementations, as shown in Appendix B. Elliptic curve scalar multiplication (ECSM) and elliptic curve double scalar multiplication (ECDSM) significantly influence ECDSA performance. Li [15] introduced a speed-oriented ECSM architecture over $GF(2^{m})$ with dual multipliers operating in parallel. Ref. [16] presents a throughput/area-efficient ECSM architecture utilizing a novel segmented digit-serial multiplier for acceleration. Khan [17] proposed a high-speed ECSM architecture employing a single multiplier and a low-latency ECSM architecture using three multipliers. These architectures modify the Lopez–Dahab Montgomery ECSM algorithm to manage data dependencies effectively. The authors of [18] introduce a flexible asymmetric crypto ECSM processor capable of handling ECSM over standard binary curves and binary huff curves. Additionally, Harb [19] presents a compact ECSM architecture tailored for small embedded applications that utilizes a ROM-based state machine to maximize hardware resource utilization.

### 1.3. Motivation

RM codes are considered highly secure in the existing literature. The security of RM codes for SRAM-PUF is contingent upon the absolute security of SRAM and the parameter ROM [20,21]. In practical scenarios, additional protective measures can be implemented to prevent attackers from reading the power-on values of SRAMs, and secure ROM can be employed to prevent attackers from reading and modifying the parameter ROM. However, these approaches entail extra hardware resources and specific electronic components. In most system-on-chip (SoC) designs, as they are modules that are directly connected to the central processing unit (CPU), conventional SRAM and ROM can be easily read and modified by attackers through software. Then, the attacker merely needs to modify $P_{error}$ in ROM based on the power-on values of SRAMs to “clone” the results of SRAM-PUF. Therefore, current research in building secure SRAM-PUFs based on conventional SRAM and ROM is still lacking, which motivates the research presented in this paper.

### 1.4. Contributions and Structure

The main contributions of this paper are as follows:

1. An RM code soft-decision attack algorithm for SRAM-PUFs is proposed. The attacker simply needs to modify the parameter $P_{\mathrm{attack}}$ in the ROM to clone the SRAM-PUF.
2. We propose a secure ECDSA SRAM-PUF based on custom signature and verification schemes. The computationally expensive modular inversion operation present in standard ECDSA is omitted in the custom schemes. The custom schemes enhance the difficulty of the proposed RM code soft-decision attack algorithm.
3. We propose a universal computing hardware architecture for ECSM and elliptic curve double scalar multiplication (ECDSM) based on the differential addition chains (DAC) to enhance the overall performance of the design.
4. A secure ECDSA SRAM-PUF architecture is proposed in this paper. The hardware architecture for RM code soft-decision emphasizes lightweight design, while the ECDSA architecture is performance-oriented. Our design is implemented on both an ASIC and FPGA to compare with the existing literature in terms of bit error rate (BER), reliability, uniqueness, and area–time product (ATP).

The remaining sections of the paper are structured as follows. Section 2 provides an introduction to the related background and preliminaries covering the fundamentals of SRAM-PUF and ECDSA. Section 3 presents the ECDSA-based SRAM-PUF scheme along with the corresponding fast algorithm. In Section 4, the paper delves into the hardware architecture of the proposed algorithm, accompanied by an exploration of hardware evaluation and optimization. Section 5 offers insights into the hardware implementation results of the proposed architecture. Finally, Section 6 serves as the conclusion, summarizing the key findings and contributions of the paper.

## 2. Security Problems Existing in Soft-Decision RM Codes

Since SRAMs are directly connected to the CPU, they are susceptible to being read by attackers through software attacks. Once SRAM is accessed by a third party, $y^{\prime }$ will be leaked to the attacker. The attacker proceeds to target the ROM, which is also directly linked to the CPU, using software methods to acquire *w* and *P*. Combining this information with $y^{\prime }$, the attacker can derive $c^{\prime }$, *c*, and *y*. The strength of PUF lies in its ability to resist replication even if an attacker gains access to the response value, as there is no means to control technological differences for modifying $y^{\prime }$. However, with the introduction of *P* through soft-decision, the paper proposes the RM code soft-decision attack algorithm, as shown in Algorithm 1. In this algorithm, the attacker can manipulate *P* to correct $c^{\prime }$ to an alternative $c^{\prime }$, thereby altering the error-corrected response value *y*.
**Algorithm 1** The Proposed Attack Algorithm for SRAM-PUFs Based on Soft-Decision RM Code**Require: *w* and *y*.****Ensure: Replicated *y*.**   1:Registration stage:   2:**for** j=0 to 100 **do**   3:   SRAMs power on to obtain one $y_{attack\_j}$;   4:**end for**   5:SRAMs power on to obtain $y_{attack}$;   6:**for** j=0 to 100 **do**   7:    **for** i=0 to $Bit\_Length(y_{attack})$ **do**   8:       **if** $y_{attack}\left[i\right]\neq y_{attack\_j}\left[i\right]$ **then**   9:          $P_{attack\_i}=P_{attack\_i}+1$;  10:      **end if**  11:    **end for**  12:**end for**  13:**for** i=0 to $Bit\_Length(y_{attack})$ **do**  14:    **if** $y_{attack}\left[i\right]\neq y_{attack\_j}\left[i\right]$ **then**  15:       $P_{attack\_i}=1-P_{attack\_i}$;  16:    **end if**  17:**end for**  18:Store *w* and $P_{attack}$ in the ROM;  19:Recovery stage:  20:SRAMs power on to obtain $y_{attack}^{\prime }$;  21:$c_{attack}^{\prime }=w⊕y_{attack}^{\prime }$;  22:$c=RM\_Decode(c_{attack}^{\prime },P_{attack})$;  23:y=c⊕w;**Return: *y*.**

In the attack scheme, a comparison is made during the registration phase between each bit of *y* and $y_{attack}$ to adjust each bit of $P_{attack}$. If a bit of *y* and $y_{attack}$ differs, the corresponding bit in $P_{attack}$ is set to $1-P_{attack}$. This adjustment ensures that the likelihood estimate for soft-decision error correction takes the opposite stance.

In the recovery phase, the disparities between $c_{attack}^{\prime }$ and $c^{\prime }$ in the attacked SRAM-PUF are analogous to those between $y_{attack}^{\prime }$ and $y^{\prime }$, given the formulas $c^{\prime }=w⊕y^{\prime }$ and $c_{attack}^{\prime }=w⊕y_{attack}^{\prime }$. Consequently, modifications to the differing bits in $P_{attack}$ between $y_{attack}^{\prime }$ and $y^{\prime }$ lead to reverse likelihood estimates for the corresponding bits. This reversal ultimately results in identical sign likelihood estimates for each bit between $c_{attack}^{\prime }$ and $c^{\prime }$. During the final decoding of the likelihood estimate to determine the symbol, two likelihood estimates with the same symbol will have a high probability of being error-corrected to yield the same code word. Thus, $c_{attack}^{\prime }$ can be corrected to $c^{\prime }$, allowing attackers to replicate *y*. As a result, attackers do not need to alter the circuits. By substituting *P* in ROM with $P_{attack}$, the same SRAM-PUF soft-decision algorithm can be ineffective at achieving the “unclonable” effect.

## 3. The Proposed Secure SRAM-PUF Scheme Based on Custom ECDSA

### 3.1. Parameter Selection for RM Code

Since the RM error correction code is used in the soft-decision algorithm, selecting the order *r* and the number *m* of the RM code is essential. Theoretically, the soft-decision algorithm can choose any RM code. However, various factors need careful consideration before the soft-decision of the RM code in PUF is made.

The order *r* dictates the recursion complexity and error correction ability of the RM code. The error correction capability of the RM code should align between the intra-chip and inter-chip error rates of the SRAM-PUF. If the error correction capability is lower than the intra-chip rate, the error correction will fail. Conversely, suppose the error correction capability surpasses the inter-chip rate. In that case, the attacker can correct the response value of the attacked chip using the response value of another chip with identical parameters, completing PUF replication. We choose r=2 so that the error correction capability of the RM code is about 20%.

The parameter *m* affects the information entropy of the RM code. Four segments of 256-bit SRAM initial values are chosen for RM(2,8) soft-decision, resulting in four responses with 37-bit information entropy each. Repeated RM code soft-decision necessitates the reuse of the same RM code soft-decision hardware multiple times, and ROM also needs to store the error probability of each segment. For different RM codes, the larger the value of *m*, the greater the information entropy of the message, but this may lead to a higher likelihood of entropy leakage caused by the code word. A value of m=8 strikes the trade-off between entropy and security. Therefore, this paper chooses RM(2,8) for generating PUF.

### 3.2. The Proposed Secure SRAM-PUF Scheme

The proposed secure SRAM-PUF scheme is illustrated in Algorithm 2. Considering the utilization of ECDSA to safeguard the error probability signature in the ROM, all error probabilities can be signed as a message during the registration stage. The algorithm of the ECDSA-PUF in the registration phase closely resembles the auxiliary data soft-decision algorithm. However, in addition to calculating *w* and *P*, it also necessitates completing the ECDSA signature.
**Algorithm 2** The Proposed Secure SRAM-PUF Scheme**Require: SRAM, a private key *d*, a public key *H*, and the order of the elliptic curve *n*.****Ensure: A stable SRAM-PUF response *y*.**   1:Registration stage:   2:**for** j=0 to 100 **do**   3:    SRAMs power on to obtain one $y_{j}$;   4:**end for**   5:SRAMs power on to obtain *y*;   6:**for** j=0 to 100 **do**   7:    **for** i=0 to Bit_Length(y) **do**   8:        **if** y[i]≠y[i] **then**   9:          $P_{i}=P_{i}+1$;  10:       **end if**  11:    **end for**  12:**end for**  13:Choose one RM code *c*;  14:w=c⊕y;  15:$\left(x_{p},s^{-1}\left(mod\;n\right)\right)=cusECDSA\_SIG\left(d,\left\{P,w\right\}\right)$;  16:$\left(X,Z\right)=cusECDSA\_VER(d,m,x_{p},NULL,s^{-1}\left(mod\;n\right))$;  17:$x_{p}Z=x_{p}\times Z\;mod\;p$;  18:Save *w*, *P*, $x_{p}Z$, $s^{-1}$ to the ROM;  19:Recovery stage:  20:$x_{p}Z=Bit\_Extension\left(x_{p}Z\right)$;  21:$w^{\prime }=w⊕x_{p}Z$;  22:SRAMs power on to obtain $y^{\prime }$;  23:$c^{\prime }=w⊕y^{\prime }⊕x_{p}Z$;  24:$c=RM\_Decode(c^{\prime },P)$;  25:$\left(X,Z\right)=cusECDSA\_VER(d,m,x_{p},x_{p}Z,s^{-1}\left(mod\;n\right))$;  26:X=Bit_Extension(X);  27:y=c⊕w⊕X.**Return: *y*.**

Once the signature is completed, the signatures are stored in the ROM. During the recovery phase, the RM code soft judgment is performed, as well as the ECDSA verification. If the verification fails, the RM code soft judgment PUF output becomes invalid. However, directly verifying the data in the ROM and using a single-bit signal to determine whether the verification passes poses a problem. This single-bit signal line directly influences the generation of SRAM-PUF, which is crucial in the circuit. Therefore, if an attacker locates this single-bit signal in the netlist or circuit layout and modifies it, the ECDSA signature verification attack can be executed, thus altering the validity of the PUF. To prevent the single-bit signal from determining the security of the entire chip, we integrate multi-bit signals throughout the algorithm. Only if the signature is verified can the correct SRAM response be obtained. Once any value in the ROM is changed, the verification will not pass, which cannot get the internal values to generate the correct response.

As the ECDSA-PUF’s signature and verification are ‘self-signed and self-verified’ as initiated by the designer, a simpler ECDSA protocol can be customized to further expedite ECDSA-PUF by omitting the time-consuming part of signature verification. Hence, the acceleration speed of the PUF is boosted. The content of the custom ECDSA protocol will not be disclosed to the public, thereby increasing the difficulty for attackers. Consequently, we propose a custom ECDSA signature cusECDSA_SIG and verification cusECDSA_VER. In the standard ECDSA, we calculate $x_{p}$ and compare it with the signature *r*. However, in the proposed custom cases, we calculate *X* and compare it with $x_{p}Z\;\left(\mathrm{mod}\;n\right)$. Hence, the time required for modular inversion is saved.

In the registration phase, power-on and power-off cycles are repeated to tally the error probability *P* and calculate the auxiliary data *w*. Subsequently, the custom signature function (Algorithm A6) is executed on *P* and *w*. Two distinctions exist between this function and the standard ECDSA signature. First, the signature *s* needs to calculate the modulo inverse $s^{-1}\;\left(\mathrm{mod}\;n\right)$. This calculation occurs in the registration phase and is conducted by computers, not during chip hardware calculation in the recovery phase. Hence, this part’s calculation is completed before leaving the factory and does not impinge on PUF generation time. Second, the function directly outputs $x_{p}$ instead of calculating $r=x_{p}\;\left(\mathrm{mod}\;n\right)$. The purpose is for the subsequent $x_{p}Z\;\left(\mathrm{mod}\;p\right)$ calculation to obtain the *P* point *X*-coordinate.

The purpose of r(modn) in standard ECDSA is to ensure *r* is smaller than the order *n*. If (modn) is skipped, it will cause r+n,r+2n,r+3n,...,r+xn to get the same sign $u_{2}$ during verification. For custom ECDSA, when $x_{p}$ serves as the signature, although $x_{p}+n,...,x_{p}+xn$ can get the same $u_{2}$ during verification, the final verification compares $x_{p}Zmodp$. Thus, (modp) will result in incorrect verification of r+xn, meaning there is no possibility that multiple numbers related to $x_{p}$ can be verified. Therefore, omitting (modn) in signatures does not affect security.

After completing the custom signature function, execute the custom verification function (Algorithm A7). The custom signature verification function directly inputs the calculated $s^{-1}\;\left(\mathrm{mod}\;n\right)$ and, ultimately, only calculates the projected coordinates *X* and *Z* without performing the modulo inverse of the coordinate transformation. After completing the signature verification and obtaining *Z*, calculate $x_{p}Z\;\left(\mathrm{mod}\;p\right)$ in advance in the registration phase, saving computation time on hardware in the recovery phase.

For standard ECDSA, *r* and *s* are safe and reliable in the ROM. For the custom ECDSA, compared with *r*, $x_{p}$ lacks the modulo step, and the amount of information in $s^{-1}\;mod\;n$ is the same as that of *s*. Thus, $x_{p}$ and $s^{-1}\;mod\;n$ are both considered safe in the ROM. In the recovery stage, we use $x_{p}Z$ to perform the auxiliary data algorithm instead of *r*. Also, perform bit expansion first, find $w^{\prime }$ and $c^{\prime }$ and perform RM code soft-decision to obtain *c*. While executing the auxiliary data algorithm, we use $x_{p}$, $x_{p}Z$, $s^{-1}\;\left(\mathrm{mod}\;n\right)$ to execute customized signature verification and calculate the projected coordinate *X* of *P*. As there is no need to perform the modular inverse step and $x_{p}Z$, $s^{-1}\;\left(\mathrm{mod}\;n\right)$ have already been calculated in the registration stage, the relevant logic for these calculation steps in the circuit of the recovery stage is saved, effectively reducing resource consumption and computation time. Finally, *X* is bit-expanded and XORed with $w^{\prime }$ and *c*. The final expression of *y* is given in Equation (1).   

(1) $$\begin{array}{cc} y=c⊕w^{\prime }⊕X & =c⊕w⊕x_{p}Z⊕X\\ & =c⊕y⊕c⊕x_{p}Z⊕X\\ & =y⊕x_{p}Z⊕X \end{array}$$

### 3.3. The Proposed Universal Algorithm for ECSM and ECDSM

For the construction of a two-dimensional DAC, we consider two scalars in ECDSM as an input vector (k,l). The two-dimensional DAC is illustrated in Appendix C. The initial values of a two-dimensional DAC are zero (0,0), the point P(1,0), and the point Q(0,1). Then, we precompute the PA results of P+Q and P−Q. Note that point subtraction is as efficient as PA since, given $Q(x_{Q},y_{Q})$, the coordinate of −Q is $x_{Q},x_{Q}+y_{Q}$. The algorithm of single PA in LD coordinates is shown in Appendix B.

**Figure A2:**
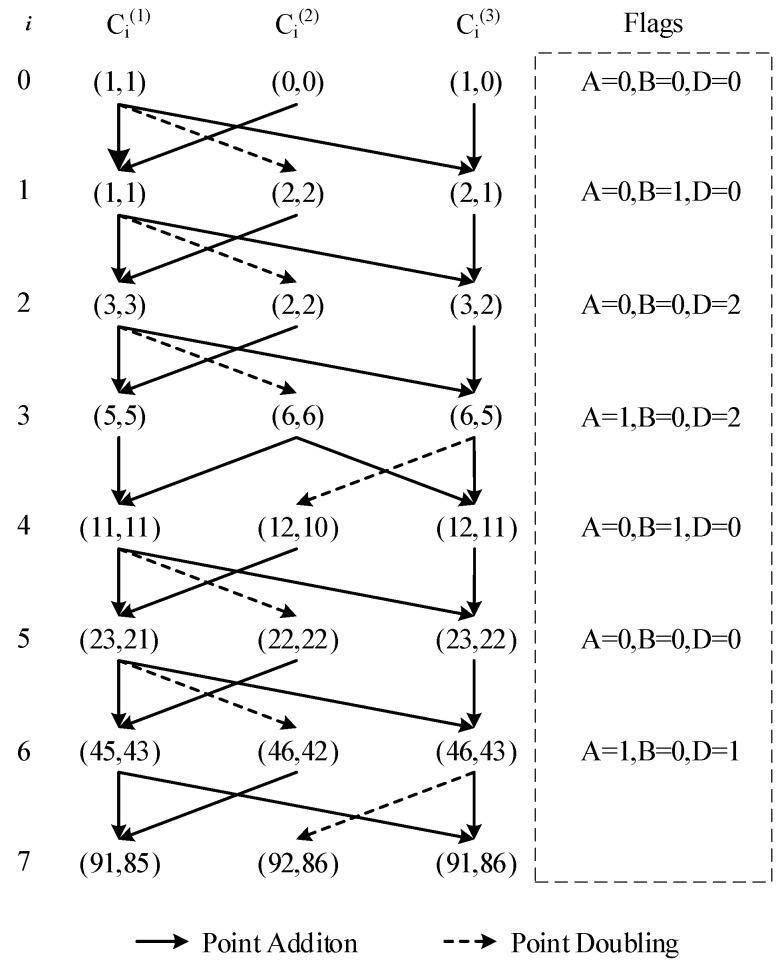
An example of ECDSM (91P+85Q) with a two-dimensional DAC.

For each loop in a two-dimensional DAC, the existence of the current vector $\left(k_{i},l_{i}\right)$ allows the calculation of four intermediate values $\left(k_{i},l_{i}\right)$, $\left(k_{i},l_{i}+1\right)$, $\left(k_{i}+1,l_{i}\right)$, and $\left(k_{i}+1,l_{i}+1\right)$. We only preserve three values out of four to reduce the computation burden. The parities of these intermediate values are (odd,odd), (even,even), (odd,even), and (even,odd). As illustrated in Figure A2, values in $C^{\left(1\right)}$ and $C^{\left(2\right)}$ are always (odd,odd) and (even,even), respectively. The missing values can be (odd,even) or (even,odd). Only one of them is preserved in $C^{\left(3\right)}$. The omitted value is determined by $\left(k_{i},l_{i}\right)$ and $\left(k_{i-1},l_{i-1}\right)$, where $\left(k_{i-1},l_{i-1}\right)=\left(\left[k_{i}/2\right],\left[l_{i}/2\right]\right)$:

1. When $\left(k_{i-1}+k_{i},l_{i-1}+l_{i}\right)=\left(odd,odd\right)$, the choice is the same as the previous iteration;
2. When $\left(k_{i-1}+k_{i},l_{i-1}+l_{i}\right)=\left(even,even\right)$, the choice is the opposite of the previous iteration;
3. When $\left(k_{i-1}+k_{i},l_{i-1}+l_{i}\right)=\left(odd,even\right)$, the choice is (even,odd);
4. When $\left(k_{i-1}+k_{i},l_{i-1}+l_{i}\right)=\left(even,odd\right)$, the choice is (odd,even).

Hence, we can generate all elements in the proposed two-dimensional DAC, as shown in Algorithm 3. Furthermore, we also need to determine the flag signals to control the accumulation for the final ECDSM results. It is obvious that $C_{i}^{\left(1\right)}$ is always calculated through the PA of $C_{i-1}^{\left(1\right)}$ and $C_{i-1}^{\left(2\right)}$. Further, $C_{i}^{\left(2\right)}$ is always calculated through the PD with one of $C_{i-1}^{\left(1\right)}$, $C_{i-1}^{\left(2\right)}$, or $C_{i-1}^{\left(3\right)}$. Finally, $C_{i}^{\left(3\right)}$ is calculated through the PA of $C_{i-1}^{\left(3\right)}$ and either $C_{i-1}^{\left(1\right)}$ or $C_{i-1}^{\left(2\right)}$. The relations in the proposed two-dimensional DAC are given as:

(2) $$\left\{\begin{array}{c} C_{i}^{\left(1\right)}=C_{i-1}^{\left(1\right)}+C_{i-1}^{\left(2\right)}\\ C_{i}^{\left(2\right)}=2C_{i-1}^{\left(n\right)}\\ C_{i}^{\left(3\right)}=C_{i-1}^{\left(3\right)}+C_{i-1}^{\left(m\right)} \end{array}\right.,$$

where n=1,2,3 and m=1,2.

To establish the two-dimensional DAC, we need to determine the data strobing for each loop. $C_{i}^{\left(1\right)}$ is always obtained through $C_{i-1}^{\left(1\right)}$ and $C_{i-1}^{\left(2\right)}$. For $C_{i}^{\left(3\right)}$, one of the adders is fixed as $C_{i-1}^{\left(3\right)}$, and the other adder can be $C_{i-1}^{\left(1\right)}$ or $C_{i-1}^{\left(2\right)}$. For $C_{i}^{\left(2\right)}$, the input of the PD can be $C_{i-1}^{\left(1\right)}$, $C_{i-1}^{\left(2\right)}$, or $C_{i-1}^{\left(3\right)}$. Meanwhile, we also need to determine the differences $x_{diff}$ between two adders within two PAs for each loop. The possible values for $x_{diff}$ can be (1,1), (0,1), (1,0), or (1,−1), which stand for P+Q, *Q*, *P*, and P−Q, respectively.

The proposed flag signal generation algorithm is shown in Algorithm 4. In this algorithm, $A_{i}$ and $B_{i}$ denote the values of *m* and *n*, respectively, in Equation (2). The difference between $C_{i-1}^{\left(1\right)}$ and $C_{i-1}^{\left(2\right)}$ can be P+Q or P−Q. When the *Y*-coordinates are omitted, the *X*-coordinates and *Z*-coordinates of P+Q and P−Q are the same. Therefore, we only determine $D_{i}$ to denote the difference between $C_{i-1}^{\left(3\right)}$ and $C_{i-1}^{\left(m\right)}$ in Equation (2).
**Algorithm 3** The Proposed Two-Dimensional DAC Generation Algorithm**Require: (k,l)****Ensure: $C_{i}^{\left(1\right)},C_{i}^{\left(2\right)},C_{i}^{\left(3\right)}$**   1:$n=max(\left\lceil log_{2}k\right\rceil ,\left\lceil log_{2}l\right\rceil )$   2:D=kmod2   3:$C_{n}^{\left(1\right)}=\left(k+\left(k+1\right)\;mod\;2,l+\left(l+1\right)\;mod\;2\right)$   4:$C_{n}^{\left(2\right)}=\left(k+k\;mod\;2,l+l\;mod\;2\right)$   5:$C_{n}^{\left(3\right)}=\left(k+\left(k+D\right)\;mod\;2,l+\left(l+D+1\right)\;mod\;2\right)$   6:**for** i=n−1 to 0 **do**   7:   Set $\left(k^{\prime },l^{\prime }\right)=\left(\left\lfloor k/2\right\rfloor ,\left\lfloor l/2\right\rfloor \right)$   8:   **if** $\left(k+k^{\prime },l+l^{\prime }\right)\;mod\;2=\left(0,0\right)$ **then**   9:         D=d  10:   **end if**  11:   **if** $\left(k+k^{\prime },l+l^{\prime }\right)\;mod\;2=\left(0,1\right)$ **then**  12:         D=0,  13:   **end if**  14:   **if** $\left(k+k^{\prime },l+l^{\prime }\right)\;mod\;2=\left(1,0\right)$ **then**  15:         D=1,  16:   **end if**  17:   **if** $\left(k+k^{\prime },l+l^{\prime }\right)\;mod\;2=\left(1,1\right)$ **then**  18:         $D=\bar{d}$,  19:   **end if**  20:   Set $C_{i}^{\left(1\right)}=\left(k^{\prime }+\left(k^{\prime }+1\right)\;mod\;2,l^{\prime }+\left(l^{\prime }+1\right)\;mod\;2\right)$,  21:   Set $C_{i}^{\left(2\right)}=\left(k^{\prime }+k^{\prime }\;mod\;2,l^{\prime }+l\;mod\;2\right)$,  22:   Set $C_{i}^{\left(3\right)}=\left(k^{\prime }+\left(k^{\prime }+D\right)\;mod\;2,l^{\prime }+\left(l^{\prime }+D+1\right)\;mod\;2\right)$,  23:**end for****Return: $C_{i}^{\left(1\right)},C_{i}^{\left(2\right)},C_{i}^{\left(3\right)}$**.

With flag signals precomputed, the proposed ECDSM algorithm is shown in Algorithm 5. $C_{0}^{\left(3\right)}$ also needs to be precomputed to determine the data strobing for the initialization of the two-dimensional DAC. For ECSM, we employed the Montgomery ladder, as shown in Appendix B. Indeed, the Montgomery ladder also involves computation through constructing a DAC, but the DAC built in the Montgomery ladder is one-dimensional. Therefore, this method does not require precomputing the parameters of the DAC. Instead, it iteratively calculates and scans each bit of *k* during the process. Moreover, whether it is the proposed two-dimensional DAC computation method or the traditional one-dimensional DAC computation method (the Montgomery ladder), the operational steps in each round of iteration are uniform (PA-PD for the Montgomery ladder and PA-PA-PD for the proposed method). Consequently, both of these computation methods can enhance the resistance against some power and timing analysis attacks. For the Montgomery ladder, each loop contains 6 multiplication operations, while our proposed ECDSM method consumes 10 multiplication operations in each loop. Hence, when executing ECDSM, our proposed method reduces the computational burden by $\frac{12-10}{12}=16.7\%$ compared to executing the Montgomery ladder twice.
**Algorithm 4** The Proposed Flag Signal Generation Algorithm**Require: $C_{i}^{\left(1\right)},C_{i}^{\left(2\right)},C_{i}^{\left(3\right)},\left(k,l\right)$****Ensure: $A_{i},B_{i},D_{i}$.**   1:$n=max(\left\lceil log_{2}k\right\rceil ,\left\lceil log_{2}l\right\rceil )$   2:**for** i=n to 0 **do**   3:    **if** $\left(C_{i+1}^{\left(2\right)}/2\right)\;mod\;2=\left(1,1\right)$ **then**   4:       Set $B_{i}=0$    5:    **else if** $\left(C_{i+1}^{\left(2\right)}/2\right)\;mod\;2=\left(0,0\right)$ **then**   6:       Set $B_{i}=1$   7:    **else**   8:       Set $B_{i}=2$   9:    **end if**  10:    **if** $\left(C_{i+1}^{\left(3\right)}\;mod\;2\;⊕\;C_{i}^{\left(3\right)}\;mod\;2\right)=\left(1,1\right)$ **then**  11:       $A_{i}=0$  12:       **if** $C_{i}^{\left(3\right)}-C_{i}^{\left(1\right)}=\left(0,1\right)$ **then**  13:          $D_{i}=0$  14:       **else if** $C_{i}^{\left(3\right)}-C_{i}^{\left(1\right)}=\left(1,0\right)$ **then**  15:          $D_{i}=1$  16:       **end if**  17:    **else if** $\left(C_{i+1}^{\left(3\right)}\;mod\;2\;⊕\;C_{i}^{\left(3\right)}\;mod\;2\right)=\left(0,0\right)$ **then**  18:       $A_{i}=1$  19:       **if** $C_{i}^{\left(3\right)}-C_{i}^{\left(2\right)}=\left(0,1\right)$ **then**  20:          $D_{i}=0$  21:       **else if** $C_{i}^{\left(3\right)}-C_{i}^{\left(2\right)}=\left(1,0\right)$ **then**  22:          $D_{i}=1$  23:       **end if**  24:    **end if**  25:    Set $k=k^{\prime },l=l^{\prime },d=D$  26:**end for****Return: $A_{i},B_{i},D_{i}$.**

**Algorithm 5** The Proposed ECDSM Algorithm

**Require: $A,B,C_{0}^{\left(3\right)},P,Q$.**


**Ensure: kP+lQ.**

   1:Set $n=max(\left[log_{2}k\right],\left[log_{2}l\right])$,   2:Set $C_{1}=P+Q,C_{2}=0$,   3:**if** $C_{0}^{\left(3\right)}=\left(0,1\right)$ **then**   4:    Set $C_{3}=Q$   5:**else if** $C_{0}^{\left(3\right)}=\left(1,0\right)$   6:    Set $C_{3}=P$   7:
**end if**
   8:**for** i=1 to *n* **do**   9:    $C_{1}\leftarrow PA\left(C_{1},C_{2}\right)$  10:    **if** $A_{i}=0$ **then**  11:       $C_{3}\leftarrow PA\left(C_{1},C_{3}\right)$  12:    **else if** $A_{i}=1$ **then**  13:       $C_{3}\leftarrow PA\left(C_{2},C_{3}\right)$  14:    **end if**  15:    **if** $B_{i}=0$ **then**  16:       $C_{2}\leftarrow PD\left(C_{1}\right)$  17:    **else if** $B_{i}=1$ **then**  18:       $C_{2}\leftarrow PD\left(C_{2}\right)$  19:    **else if** $B_{i}=2$ **then**  20:       $C_{2}\leftarrow PD\left(C_{3}\right)$  21:    **end if**  22:
**end for**


**Return: kP+lQ.**



## 4. Hardware Architecture

### 4.1. The Overall Architecture of ECDSA SRAM-PUF

**Figure 1:**
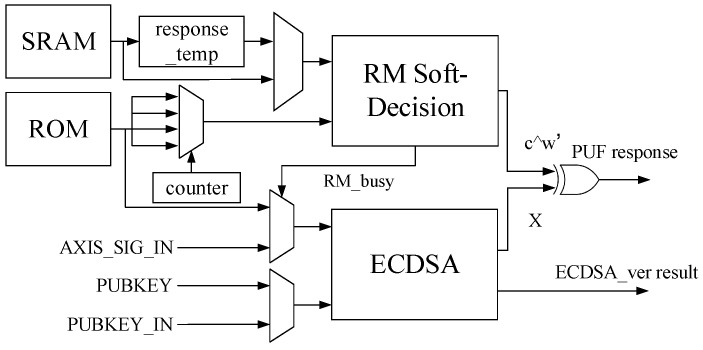
The overall structure of ECDSA-PUF.

The hardware implementation of ECDSA-PUF is illustrated in Figure 1, where the algorithm integrates ECDSA and RM code soft-decision. The primary hardware components consist of the ECDSA module and the RM code soft-decision module. The XOR operation combines the signature and the RM code, generating the SRAM-PUF. Notably, during placement and routing, there is no distinct boundary or single-bit key signal between the modules. The ECDSA module and the RM code soft-decision module are the core hardware components of ECDSA-PUF, and their detailed hardware structures will be elucidated in subsequent subsections.

The overall hardware structure of ECDSA-PUF comprises two core algorithm modules, the ECDSA module and the RM code soft-decision module, along with several multiplexers. The ECDSA module not only performs ECDSA-PUF signature verification but also functions as a system peripheral for the system’s ECDSA signature verification request. With the idea of multiplexing, the ECDSA module supports both the custom ECDSA signature verification proposed in this paper and the standard ECDSA signature verification. The input passes through a selector to RM_busy, serving as the sel signal for the selector. When RM_busy is low, the signature and public key on the input bus complete standard ECDSA verification. Conversely, when RM_busy is high, the signature in the input ROM and the fixed public key in the circuit complete custom ECDSA signature verification.

The public key of ECDSA-PUF must be consistently fixed within the circuit to maintain a constant value. This measure ensures that only this specific public key is employed for ECDSA-PUF signature verification. Failure to secure the fixed public key in the circuit could expose vulnerabilities. Therefore, an attacker may substitute the ECDSA public key in the PUF with an alternative private–public key pair to launch an attack potentially. The ECDSA module operates in distinct working modes, resulting in different outputs. During ECDSA-PUF calculation, it produces the output *X*, which is then XORed with the output result $c⊕w^{\prime }$ of the RM code to derive the response *y*. Following the successful generation of the PUF response, ECDSA-PUF ceases operation. Mechanisms such as gated clocks or power gating can be employed to deactivate the clock or power supply of the RM code soft-decision module to conserve power consumption. Concurrently, RM_busy remains consistently at zero, and the ECDSA module transitions to performing the standard ECDSA task of the system, ultimately outputting the verification result (pass or fail).

In addition to algorithm modules and multiplexers, there are memory modules in the structure to cache initial values and intermediate variables. In the overall hardware system, three data storage units are used: SRAM, response_temp, and ROM.

1. SRAM: Provides the initial response $y^{\prime }$ of the PUF. The data in it become invalid after the response is read, and it is then used as the data RAM to cache the intermediate variable of the RM code.
2. response_temp: Caches the initial value of SRAM. To improve the entropy of PUF, four segments of 256-bit SRAM initial values are selected and repeated four times to obtain a 1024-bit response. The RM code structure needs to be multiplexed and calculated four times. After a calculation is completed, the initial value in the SRAM will be overwritten by the intermediate result of the calculation. To preserve the remaining initial values of the SRAM, response_temp is added to cache the other three initial values of the SRAM.
3. ROM: Stores the *w* and *P* of the auxiliary data algorithm as well as the signature values $x_{P}$, $x_{P}Z$, $s^{-1}\;\left(\mathrm{mod}\;n\right)$ required for signature verification. The variables *w* and *P* need to output the value corresponding to the multiplexing times when calculating the RM code for each multiplexing.

### 4.2. The Architecture of Soft-Decision RM Code

**Figure 2:**
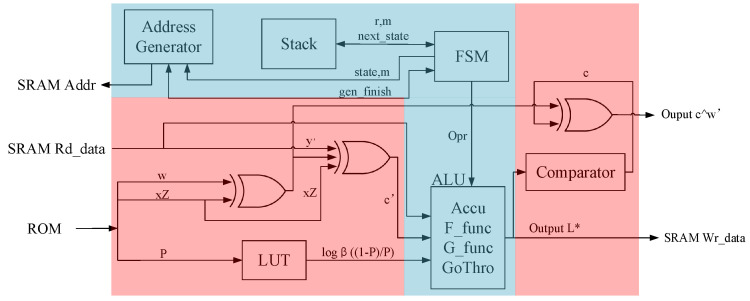
RM code soft-decision architecture.

The RM code soft-decision module is one of the core algorithm modules of the ECDSA-PUF hardware. Its hardware structure is shown in Figure 2. The auxiliary calculation architecture (red part) is responsible for executing the recovery stage in Algorithm 2. In contrast, the RM decoding architecture (blue part) is responsible for executing the recursive algorithm as shown in Algorithm A3.

The auxiliary calculation architecture comprises core components such as two XOR gates at the input of the arithmetic logical unit (ALU), a comparator with an XOR gate at the output of the ALU, and a look-up table (LUT) for calculating likelihood estimation. These components serve various functions in the recovery phase of Algorithm 2.

1. XOR gates: Two XOR gates at the input of the ALU are utilized to perform XOR operations during the recovery phase. Specifically, they are involved in XORing operations for obtaining $w^{\prime }$ and $c^{\prime }$.
2. Comparator with XOR gate: The comparator, coupled with an XOR gate at the ALU’s output is responsible for converting the likelihood estimation obtained after calculations into a code word *c*. The XOR gate in this context contributes to the computation of $c^{\prime }$.
3. LUT: The LUT is employed to calculate the likelihood estimation using the formula $\log_{\beta }\frac{1-P}{P}$. Hardware implementation of this logarithmic calculation can be complex. However, due to the limited set of possible error probability values *P* derived from 100 instances of power on and off during the registration phase, a pre-calculated LUT is used. The LUT helps obtain the corresponding $\log_{\beta }\frac{1-P}{P}$ for each *P*, significantly saving computational time. Additionally, the LUT’s corresponding relationship can be randomized to enhance the difficulty of attacker interference.

The data path of the auxiliary calculation architecture follows these steps: SRAM outputs the response $y^{\prime }$, and ROM outputs *w*, $x_{P}Z$, and *P*. The XOR operation between *w* and $x_{P}Z$ yields $w^{\prime }$, and the XOR operation involving $y^{\prime }$, $w^{\prime }$, and $x_{P}Z$ results in $c^{\prime }$. Subsequently, $c^{\prime }$ combines with $\log_{\beta }\frac{1-P}{P}$ to form the likelihood estimate value *L*. This value is input into the ALU to execute the recursive algorithm. Upon completion of the recursive algorithm, $L^{*}$ is output, and the correct codeword *c* is obtained after error correction through the comparator. Finally, performing XOR with $w^{\prime }$ and the outer-layer signature verification result *X* yields the correct PUF response *y*.

The RM decoding architecture comprises essential components such as the ALU, an address generator, a stack, and a state machine. Each component plays a unique role in the overall function of the recursive module. Here is a breakdown of their roles:

1. ALU: Performs calculations related to the F function, G function, and accumulation, as specified in Algorithm A3. Facilitates operations such as passing data directly to the next module.
2. Address generator: Generates the current corresponding SRAM read and write addresses based on the algorithm’s requirements.
3. Stack: Functions as a cache unit that stores parameters and local variables for each round of recursion. Enables the implementation of a software-driven approach to realize hardware recursion. This approach reduces the complexity of the state machine by offloading certain control aspects to the stack.
4. State machine: Serves as the core control logic for the entire recursive module. Utilizes a software-driven approach, allowing certain steps of the recursion to be expanded into a large state machine. Manages the control of the current recursive round, while the recursion of other rounds is controlled by parameters stored in and retrieved from the stack.

**Figure 3:**
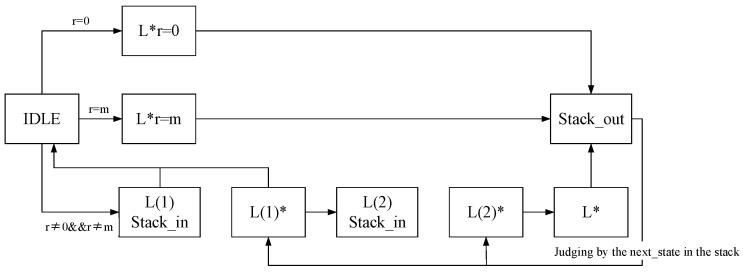
RM code soft-decision state machine.

The data path of the recursive algorithm is illustrated in Figure 3. The likelihood estimate value *L* computed by the auxiliary data algorithm serves as the input to the ALU, initiating the recursion process, which is governed by the state machine. During the calculation state, the ALU performs the corresponding calculations, and the results are output into the SRAM cache. Subsequent iterations retrieve the SRAM data, which are then returned to the ALU for further computations. In the jump state, the ALU transfers data from one address in the SRAM to another. The cycle of transitioning between the calculation state and the jump state continues until the soft-decision of the RM code is completed, ultimately outputting $L^{*}$.

The states involving pushing and popping the stack—namely, L(1) pushing the stack, L(2) pushing the stack, and popping the stack—serve as jump states responsible for the logic control of the recursive algorithm. In the pushing states, if the current execution transition corresponds to pushing the stack, the parameters of the current recursion round (next_state, *r*, *m*) are saved and pushed into the stack. Subsequently, the state machine restarts from the IDLE state, initiating a new round of recursion. Conversely, in the state of popping the stack, the state machine reads the saved parameters (next_state, *r*, and *m*) from the previous round of recursion in the stack. The machine then continues the recursion until the entire recursive process is completed.

The five states, $L_{r=0}^{*}$, $L_{r=m}^{*}$, $L{\left(1\right)}^{*}$, $L{\left(2\right)}^{*}$, and $L^{*}$, belong to the calculation state and correspond to the computation of the variables in Algorithm A3. These states entail the accumulation of the F function, G function, and SoftDecision_Decode_Rep function. The ALU performs these calculations and selects the current operation through the opr signal associated with the current state.

The speed of the RM code soft-decision relies on the amount of SRAM read and write operations, making it essential to evaluate the hardware speed based on these factors. Therefore, an analysis of the data and address allocation of the SRAM is conducted. In each state of the state machine, the addresses and lengths of SRAM read and write operations vary depending on the current *m* value and state. Consequently, the address generator module generates SRAM-accessible addresses corresponding to the current state of the state machine and the current *m* value.

The states $L^{\left(1\right)}$ and $L^{(2)*}$ exhibit no data dependency under the same round of recursion, enabling them to share the same storage space. With SRAM being 32 bits wide, the selected bit width for *L* and each intermediate variable has a 16-bit length, ensuring sufficient data precision without risking overflow. When mapping addresses, two adjacent 16-bit segments are placed into one address. For m=8, the required SRAM space for the RM code soft-decision hardware is 512×4×2 Bytes = 4 KB. Following the SRAM analysis, the soft-decision speed of the RM code can be calculated based on the data volume in the SRAM. For each *r*, there are 20 reads and writes, resulting in 512×20=10,240 cycles for all *m*. Since 256+128+64+…=512 groups for all *m*, the total number of SRAM reads and writes required for the entire recursion is 512×20=10,240 cycles. Completing four soft-decisions of the RM code takes 10,240×4=40,960 cycles. Factoring in the extra time for other logic, the total required cycles amount to approximately 41,000 cycles.

### 4.3. The Architecture of ECDSA

**Figure 4:**
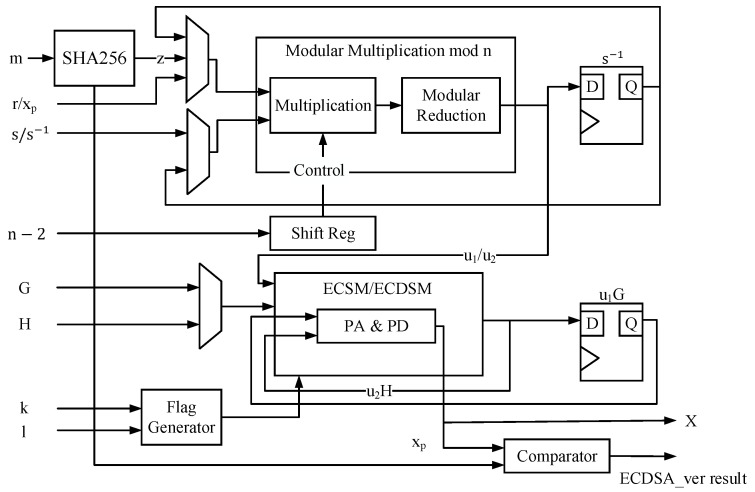
The hardware architecture of ECDSA.

The ECDSA module constitutes another core algorithmic component of the ECDSA-PUF hardware. Its hardware architecture is illustrated in Figure 4. Diverging from numerous existing ECSM architectures in the preceding chapters, the proposed ECDSA module ascends from the group operation layer to the ECDSA protocol layer. Consequently, beyond the ECSM/ECDSM module, it becomes imperative to accomplish the SHA256 module and the modular multiplier of the order *n* mandated by ECDSA to fulfill the complete ECDSA protocols.

The ECDSA architecture is designed to perform two distinct types of signature verification. It executes cusECDSA_VER during the PUF generation stage and conducts the standard ECDSA signature verification algorithm as a hardware acceleration peripheral of the system after the PUF is generated. Therefore, this architecture must embody the concept of hardware reuse by utilizing a universal ECSM/ECDSM module and a modular multiplier with different calculation methods based on the specific calculation mode. Hardware multiplexing introduces additional multiplexers to govern the data paths in other modes. Our design allows the utilization of the same large-number multipliers and large-number adders for the underlying operations in various modes to conserve hardware resources.

The data path of the ECDSA architecture follows these steps:

1 The value *m* is processed in the SHA256 module through the hash operation to derive the digest value of the message. A fixed random interception is employed to capture 163 bits. It is crucial to note that the random interception must be firmly embedded in the circuit to prevent potential manipulation by attackers attempting to alter the HASH value through the configuration of the interception position. The hash interception may result in entropy loss; hence, entropy augmentation is performed in subsequent steps to restore the entropy that has been lost.
2 Compute $u_{1}$ in the signature verification algorithm. In the context of cusECDSA_VER, the values $s^{-1}$ and $x_{P}$ directly feed into the modular multiplier for the computation of $u_{1}$. In contrast, during standard ECDSA signature verification, the value *s* requires modulo inverse calculation using Fermat’s little theorem. Hence, a shift register is incorporated in the structure to sequentially output each bit of n−2, controlling the input to the modulo multiplier and buffering the intermediate result in the $s^{-1}$ register.
3 Calculate the ECSM of $u_{1}$ by the base point *G*. The result of the ECSM is cached in the $u_{1}G$ register. According to the pipeline idea, the modular multiplier calculates $u_{2}$ simultaneously, and the calculation of $u_{2}$ is similar to the calculation of $u_{1}$. Since $s^{-1}$ is already obtained when calculating $u_{1}$, performing a modular inversion is unnecessary.
4 The output of the modular multiplier transitions from $u_{1}$ to $u_{2}$. Subsequently, the strobe signal of the input selector is altered to input the public key *H*, initiating the ECSM of the public key *H* by $u_{2}$.
5 Compute the sum of $u_{1}G$ points and $u_{2}H$, generating distinct outputs based on the ECDSA module. For standard ECDSA signature verification, compute $x_{P}$; for cusECDSA_VER, compute *X*.
6 For standard ECDSA signature verification, compare $x_{P}$ with *r*. The module outputs 1 for identical results and 0 for different ones. For cusECDSA_VER, the module obtains four *X* values after four iterations. These 163-bit *X* values need to be extended to four 256-bit *X* values to match the number of RM code soft-decisions and restore the lost entropy. The extension method must use the same approach as the extension of $x_{P}Z$ stored in the ROM; otherwise, the same value cannot be obtained after the expansion of *X* and $x_{P}Z$, preventing completion of the signature verification due to failure to satisfy Equation (1).

**Figure 5:**
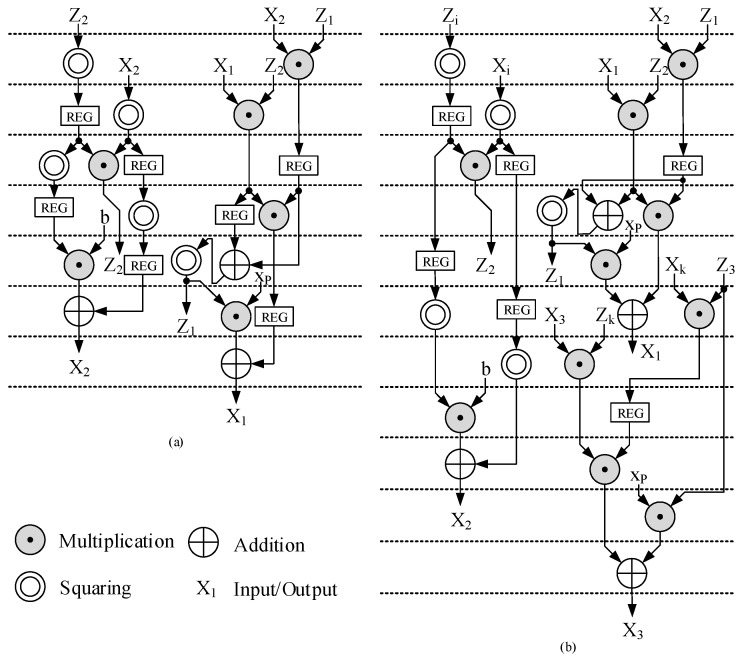
The proposed timing schedule of (**a**) ECSM and (**b**) ECDSM.

Based on the data dependency introduced by Equations (A9) and (A10), we proposed the timing schedule for ECSM and ECDSM with one two-stage multiplier and one square unit as shown in Figure 5a,b. “REG” represents a register in this clock cycle buffering the current intermediate value.

The timing schedule for ECSM is based on the Montgomery ladder Algorithm A4. With one two-stage multiplier, we proposed a compact six-clock-cycle (6 CC) timing schedule, as shown in Figure 5a. The multiplier is always busy, leaving no idle clock cycle. Based on this compact schedule, it consumes 6 CCs for each loop. Note that the figure illustrates the calculation process over seven clock cycles, where the seventh is also the next iteration’s first cycle. The timing schedule for ECDSM follows a similar pattern. In this timing schedule, the modular multiplication operations of PD, $Z_{2}=X_{2}^{2}Z_{2}^{2}$ and $X_{2}=bZ_{2}^{4}+X_{2}^{4}$, locate at clock cycles 3–4 and clock cycles 5–6, while other multiplication operations belong to PA. The squares are arranged as close to the related multiplication as possible to avoid wasting registers for holding internal values.

The timing schedule for ECDSM follows the proposed Algorithm A4. Each loop involves two PAs and one PD, resulting in 10 multiplication operations per loop. Based on this design philosophy, we introduce a compact ECDSM timing schedule utilizing a two-stage multiplier and a square unit, as illustrated in Figure 5b. Ten CCs are required to execute two PAs and one PD in each loop. The values of *i* and *k* are determined by the proposed flag signal generation Algorithm 4 during precomputation. After precomputation, modular multiplications of PD are scheduled at clock cycles 3–4 and 8–9, enabling the first PA in ECDSM to advance by one clock cycle. Meanwhile, the second PA remains idle during clock cycles 8–9 to wait for the results of $X_{3}Z_{k}$; thus, another multiplication of PD is scheduled at clock cycles 8–9 to ensure an utterly compact timing schedule without any wasted clock cycles.

The two-stage multiplier employs the Karatsuba–Ofman algorithm, with carefully inserted pipeline stages to alleviate critical paths. Two squaring units are directly cascaded, requiring one clock cycle for square and quartic powering operations. During ECDSM computation, the DAC generator precomputes chain parameters based on scalars *k* and *l*. Additionally, a finite-state machine governs the operational modes of the architecture. The register bank includes extra registers for storing internal values during ECSM and ECDSM operations. Each register is connected to a multiplexer to regulate the datapath. Control signals from these multiplexers are consolidated into instructions executed at every clock cycle.

## 5. Implementation Results

### 5.1. ASIC Results

The proposed secure ECDSA SRAM-PUF architecture can be implemented using an FPGA or an application specific integrated circuit (ASIC). With the same FPGA platform as used in existing research, we can conduct a fair comparison. Note that fairness is not assured in ASIC comparisons due to significant performance variations of the same circuit for different processes, voltages, and temperatures. However, the PUF is strictly related to hardware and cannot be reliably verified by a FPGA. Further, many existing PUF designs are based on ASIC implementations. Therefore, our proposed architecture is implemented on both an FPGA and an ASIC. For the ECDSA part, we primarily compare the FPGA implementation results with existing designs to ensure fairness in the comparison. Regarding the PUF implementation results, we aim to provide performance metrics for our designed architecture compared to existing designs for the readers’ reference.

The ASIC results are synthesized using the SMIC 40 nm library with the Synopsys design compiler. The system achieves a maximum clock frequency of 400 MHz. The total area is 327,533.2 μm^2^, with the PUF consuming 15,776.6 μm^2^. This result is before place and route. The gate equivalent (GE) is calculated as the total area divided by the area of one NAND2 gate under the corresponding process.

The hardware implementation of the fast ECDSA-PUF algorithm is compared with other works on SRAM-PUFs. This comparison focuses primarily on achieving a lower bit error rate (BER) and GE per bit. BER measures PUF stability, while GE per bit reflects hardware resource consumption independent of PUF length. GE per bit can be calculated using Equation (3), as proposed in [22]. Both the BER and GE per bit parameters are relatively unaffected by the process. Our design is a 1024-bit PUF; the comparison results are listed in Table 1.

(3) GEperbit=area/NAND2area/PUFbit

Table 1 indicates that the GE per bit of our design falls within the mid-range. Notably, refs. [23,25] demonstrate lower GE per bit values but suffer from higher BER, potentially affecting chip functionality. Moreover, the proposed ECDSA-PUF hardware architecture increases the SRAM-PUF bit count by utilizing multiplexing within the ECDSA-PUF module. This is achieved through the repetition of custom ECDSA and soft-decision calculations. Consequently, the GE per bit of the proposed design would decrease significantly in scenarios requiring greater information entropy. This scalability is not achievable in other related works, highlighting the advanced nature of the proposed design in terms of lightweight characteristics. While the SRAM-PUF proposed by [22] excels in BER, the fast ECDSA-PUF presented in this paper prioritizes speed, security, and lightweight attributes. Thus, an error correction code with a lower BER is deemed sufficient for prevailing BER requirements.

Our fabricated chips in the 40 nm process were evaluated at room temperature of 25 °C with the standard supply voltage (1.0 V) to measure the intra-chip variation and inter-chip variation. To measure intra-chip variation, we compared the response to the same challenge on the same chip 1000 times. To ensure the experiment’s reliability, we derived the results of intra-chip variation by measuring five chips rather than one single chip. The intra-chip variation of our design has a mean value (M) of 49.44% with 2.44% standard deviation (SD). Further, we measured the output responses of all our chips (40 chips) with the same challenge to measure inter-chip variation. The results of inter-chip variation are M = 49.44% and SD = 2.44%, and the mean value of our design is close to the ideal value (M = 50%). The quality result of SRAM-PUF in our design is compared with existing research in Table 2.

To measure the temperature resistance and voltage resistance of the SRAM-PUF, we conducted BER measurements on the SRAM-PUF in our design across a temperature range from 0 °C to 85 °C and a voltage range from 0.8 V to 1.2 V (±20% of the standard voltage). The most error-prone case arises when the temperature and voltage are 85 °C and 1.2 V, respectively. In this case, the BER reaches 11.21%, approaching the error correction capability upper limit of the RM(2,8) in our design. In this extreme scenario, we conducted 100 complete experiments for each chip, and no response errors occurred.

### 5.2. FPGA Results

The proposed architecture was instantiated on the Xilinx Virtex-7 FPGAs using Vivado 2022, a choice made to ensure a fair and contemporary comparison with existing designs. To gauge our design’s performance in a manner that is both comprehensive and reasonable, we executed the architecture across NIST-recommended $GF(2^{163})$ and encompassed variations in scale to provide a thorough evaluation of its capabilities and efficiency. Considering the ECDSA in our work is a custom design, we compare the performance of ECDSM with existing works to ensure fairness in comparison. For existing designs that only implement ECSM, we consider twice their total latency as an approximate latency for ECDSM. In reality, this rough evaluation method often yields more optimistic estimates for existing designs, as it neglects the PA after two ECSMs.

In our design, the total latency includes DAC generation, iteration, and inversion. Although in the precomputation stage, our design needs to compute both flag signals for DAC construction and P±Q, these two parts are executed in separate circuit components, allowing them to be performed in parallel. Moreover, the latency of computing flag signals is significantly greater than computing P±Q. Therefore, in the total latency consideration, we no longer account for the latency introduced by P±Q. For $GF(2^{m})$, constructing the DAC chain requires *m* clock cycles. The total latency can be calculated by Equation (4).

(4) $$T=\left(C_{DAC}+C_{ITR}+C_{INV}\right)\times T_{CLK}$$

In our design, there is one multiplier and one square unit. When utilizing Itoh’s [30] proposed modular inversion algorithm, the modular inverse can be calculated within m+1 cycles. The iteration consumes 10×m+1 clock cycles over GF($2^{m}$) and one additional clock cycle to wait for the final multiplication result.

(5) $$C_{ITR}=m+10\times m+\left(m+1\right)+1$$

Most existing research utilizes ATP as a performance benchmark to assess the trade-offs between hardware resources and latency. While some research evaluates the area using the number of LUTs #LUT, most employ the number of slices #Slice. In our design, we evaluate ATP using the number of slices to ensure fair comparisons, as our design introduces additional storage resources. We estimate the number of slices for the literature lacking slice data based on reported LUT data. For Xilinx Virtex-7 FPGAs, each slice typically contains four LUT6. However, not all utilized slices are fully occupied with all four LUT6. Therefore, the ratio of LUTs to slices, $\frac{\#LUT}{\#Slice}$, is typically between 3 and 3.5. We set this ratio to 3.5 in this paper to facilitate fair comparisons.

(6) ATP=#Slices×T.

Table 3 shows the results comparison of ECDSM over $GF(2^{163})$. Due to adopting the DAC for ECDSM calculation, the number of clock cycles required for our design is significantly lower than those of existing designs using a single multiplier. From the perspective of area–speed trade-off, our design achieves a better ATP indicator, being on par with existing designs [15,16]. This design’s latency is inferior to [15,17], as both [15,17] adopt architectures with multiple multipliers. Hence, the number of clock cycles needed to compute ECDSM is minimal. However, at the same time, the area cost of existing designs [15,17] is larger: especially design [17], which uses three parallel multipliers. From the area–speed trade-off perspective, this design’s ATP surpasses existing designs [15,17].

The PUF on the FPGA has a total cost of 112 slices, which is very limited compared to the cost of ECDSA. The PUF in this design is used only during the system startup phase and not during regular system operation, so a miniature PUF can effectively save hardware resources. The ECDSA, ECSM, and ECDSM functions in our designs can also be reused for other purposes. Therefore, we have consumed most of the logic resources to build a high-performance ECDSA architecture.

## 6. Conclusions

This paper proposes a universal ECSM/ECDSM architecture for constructing the secure ECDSA SRAM-PUF presented herein. Initially, the paper outlines an attack scheme for PUFs constructed from conventional SRAM and ROM within SoCs. This scheme demonstrates that by repeatedly powering the system on and off and exploiting the SoC’s processor to access SRAM and ROM to tamper with the *p* value, the PUF’s unclonability can be compromised. This paper leverages the ECDSA to counteract this attack scheme and designs an SRAM-PUF architecture based on a custom ECDSA, ensuring the *p* value remains untampered with through the custom ECDSA. The paper proposes a universal architecture for critical operations, such as ECSM and ECDSM. For ECSM calculations within ECDSA, iterations of PA and PD can be performed directly; for the more time-consuming ECDSM calculations within ECDSA, a two-dimensional DAC is constructed through precomputation, followed by iterations of PA and PD based on the two-dimensional DAC. The ECDSM based on a two-dimensional DAC theoretically saves 16.7% of the computational overhead compared to executing ECSM twice, significantly increasing computation speed. Moreover, this universal architecture saves a significant amount of hardware resources due to the high similarity in the datapaths of ECSM and ECDSM. The secure ECDSA SRAM-PUF proposed in this paper is implemented on ASIC and FPGA. This design exhibits lower BER and better ATP performance compared to existing research. In the future, we will further exploit DAC-based ECDSA over GF(p).

## Figures and Tables

**Table 1:** PUF implementation results comparison.

Research Work	ASIC Process (nm)	Area Per Bit (μm^2^/bit)	GE Per Bit (GE/bit)	BER
[22]	65	49.12	25.58	$2.7\times 10^{-10}$
[23]	14	1.84	11.83	$1.45\times 10^{-2}$
[24]	65	50.7	26.40	$1.56\times 10^{-2}$
[25]	65	17.9	9.32	$2.5\times 10^{-2}$
This work	40	15.40	21.45	$3\times 10^{-5}$

**Table 2:** PUF quality comparison.

Research Work	Device or Process	Reliability Intra-Chip Variation	Uniqueness Inter-Device Variation	Temperature	Voltage Supply
Latch-PUF [26]	Spartan-3E	M = 2.4% SD = 0.75%	M = 46% SD = 3.8%	0 °C–85 °C	— *
Latch-PUF [26]	Spartan-6	M = 0.86% SD = 0.54%	M = 49% SD = 3.9%	—	1.14 V–1.26 V
SRAM-PUF [27]	45 nm	M = 0.72% SD = 10%	M = 49.97% SD = 15%	10 °C–85 °C	—
Butterfly-PUF [28]	65 nm	$M_{max}$ = 6% —	M = $50_{around}$% —	−20 °C–80 °C	—
TERO-PUF [29]	Cyclone II	M = 1.7% —	M = 48% —	28 °C	1.5 V
Delay-Hardened-PUF [23]	14 nm	M = 3.4% —	M = 48.6% —	25 °C–110 °C	0.55 V–0.75 V
Amplifier-PUF [25]	180 nm	M = 0.07% SD = 0.32%	M = 49.89% SD = 6.24%	−40 °C–120 °C	0.8 V–1.8 V
This work	40 nm	M = 3.17% SD = 1.63%	M = 49.44% SD = 2.44%	0 °C–85 °C	0.8 V–1.2 V

* Not reported in the literature.

**Table 3:** FPGA implementation results on Vertex-7 series over $GF(2^{163})$.

Design	Clock Cycle	Freq.	#LUT	#Silce	Latency	ATP
[15]	547	320.5	28,911	8460	3.413	28,878
[16]	4168	397	4271	1476	20.997	30,992
[17]	450	159	41,090	11,657	5.660	65,983
[31]	780	223	27,105	8736	6.995	61,113
[32]	3960	369	9965	2207	21.463	47,370
[18]	3960	351	10,955	3107	22.564	70,107
[19]	13,000	320.8	6169	2201	81.047	178,385
[33]	52,012	800	− *	4665	130.03	606,590
[34]	3426	135	−	3657	50.076	185,613
This Work	1958	296	13,912	3902	6.615	25,812

* Not reported in the literature.

**Figure A1:**
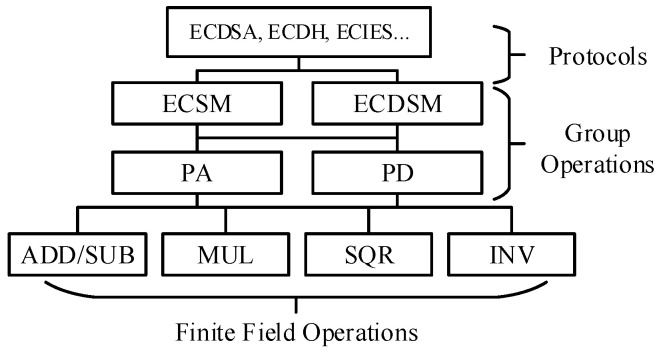
The three-level hierarchy of ECC.

## Data Availability

All data can be provided upon reasonable request to the corresponding author.

## Appendix A. Soft-Decision SRAM-PUF Based on Reed–Muller Codes

The algorithm of soft-decision SRAM-PUF based on Reed–Muller codes is given in Algorithm A1, which contains the registration stage and the recovery stage.

1. Registration stage: Initiate the SRAM power cycle, toggling it on and off for 100 iterations to document error probabilities $P_{i}$. These probabilities indicate the likelihood of each bit differing from the corresponding bit in the standard value *y*. The recorded error probabilities serve as indicators for the forthcoming response generation, revealing the likelihood of each bit being the same or different from the corresponding bit in *y*. Subsequently, randomly select an RM code *c* and calculate the auxiliary data *w* by an exclusive-OR operation (XOR). Then, the auxiliary data *w* and error probabilities *P* are both stored in the ROM. The registration stage must be concluded before the chips depart the factory. The statistical analysis of *P* and the storage of *w* and *P* are stored during the manufacturing or testing phases of the chip.
2. Recovery stage: Following transactions, customers receive a response $y^{\prime }$ with an error code when the SRAM is powered on. The value $c^{\prime }$ is obtained by XORing with *w*. Subsequently, utilizing RM code soft-decision decoding, $c^{\prime }$ and error probability *P* are inputted to execute error correction. The error probability *P* enhances error correction capabilities by providing additional information to the RM code. Following the correction, the corrected code *c* is obtained, and c⊕w yields the SRAM-PUF response *y*. This stage is completed on the chip by customers.

The RM code is a high-order repeat code obtained through iterative recursive operations of repeat codes. Therefore, the soft-decision method for RM codes evolves from the soft-decision method of repeat codes through recursive calculations. The soft-decision for repeat codes involves calculating the likelihood estimate $L_{i}$ for each bit by substituting the bit error probability into Equation (A1). In this formula, β is a constant adjusted for different precisions, and $P_{i}$ and $c_{i}^{\prime }$ represent the error probability for each bit and the codeword, respectively. The “majority selection” is employed for repeat codes, where the result of the majority bits is considered the correct value. The likelihood estimates for each bit are summed to obtain the overall likelihood estimate $L^{*}$ for the codeword. As shown in Algorithm A2, if $L^{*}>0$, the probability of the repeat code codeword *c* being all zeros is greater, resulting in a decoded result of all zeros. Conversely, $L^{*}<0$ indicates that the probability of *c* being all ones is greater, leading to a decoded result of all ones.

(A1) $$L_{i}={\left(-1\right)}^{c_{i}^{\prime }}\log_{\beta }\frac{1-P_{i}}{P_{i}}.$$

**Algorithm A1** Soft-Decision SRAM-PUF Algorithm Based on Reed–Muller Codes
**Require: SRAMs.** **Ensure: A stable response *y*.** 1: Registration stage: 2: **for** j=0 to 100 **do** 3: SRAMs power on to obtain $y_{j}$; 4: **end for** 5: SRAMs power on to obtain *y*; 6: **for** j=0 to 100 **do** 7: **for** i=0 to Bit_Length(y) **do** 8: **if** $y\left[i\right]\neq y_{j}\left[i\right]$ **then** 9: $P_{i}=P_{i}+1$; 10: **end if** 11: **end for** 12: **end for** 13: Choose one RM code *c*; 14: w=c⊕y; 15: Store *w* and *P* in the ROM; 16: Recovery stage: 17: SRAMs power on to obtain $y^{\prime }$; 18: $c^{\prime }=w⊕y^{\prime }$; 19: $c=RM\_Decode(c^{\prime },P)$; 20: y=c⊕w; **Return: *y*.**

**Algorithm A2** Soft-Decision Repeat Code
**Require: *L* and *n*.** **Ensure: $L^{*}$.** 1: SoftDecision_Decode_Rep(L,n); 2: $L^{*}=\sum_{i=1}^{n}L_{i}$; **Return: $L^{*}$.**

The RM code encompasses two parameters: the order *r* and the frequency *m*, where *r* determines the recursion complexity, and *m* determines the length of the code word $2^{m}$. An RM (*r*, *m*) code can be decomposed into an RM (r−1, m−1) code and an RM (*r*, m−1) code. When r=0, the RM code becomes a repetitive code, and when r=m, the RM code lacks error correction capability. For RM codes, the decoding processing is similar to repeat codes. Firstly, calculate the likelihood estimate *L* for each bit based on Equation (A1). Subsequently, substitute the likelihood estimates into Algorithm A3 for recursion, ultimately obtaining the likelihood estimate $L^{*}$ for each bit after decoding.

**Algorithm A3** Soft-Decision RM Code
**Require: *L*, *r*, and *m*.** **Ensure: $L^{*}$.** 1: Define F(a,b)=sign(a×b)×min(|a|,|b|); 2: Define $G\left(s,a,b\right)=\lfloor \frac{1}{2}\left(sign\left(s\right)\times a+b\right)\rfloor$; 3: SoftDecision_Decode_RM(L,r,m); 4: **if** r=0 **then** 5: $L^{*}=SoftDecision\_Decode\_Rep\left(L,2^{m}\right)$; 6: **else if** r=m **then** 7: $L^{*}=L$; 8: **else** 9: $L_{i}^{\left(1\right)}=F\left(L_{2i-1},L_{2i}\right),i=1,\ldots ,2^{m-1}$; 10: $L^{(1)*}=SoftDecision\_Decode\_RM\left(L^{\left(1\right)},r-1,m-1\right)$; 11: $L_{i}^{\left(2\right)}=G\left(L_{i}^{(1)*},L_{2i-1},L_{2i}\right),i=1,\ldots ,2^{m-1}$; 12: $L^{(2)*}=SoftDecision\_Decode\_RM\left(L^{\left(2\right)},r,m-1\right)$; 13: $L^{*}=\left(F\left(L_{1}^{(1)*},L_{1}^{(2)*}\right),L_{1}^{(2)*},\ldots ,F\left(L_{2^{m-1}}^{(1)*},L_{2^{m-1}}^{(2)*}\right),L_{2^{m-1}}^{(2)*}\right)$; 14: **end if** **Return: *X* and *Z*.**

## Appendix B. Elliptic Curve Cryptography Arithmetic

ECDSA consists of two stages: the signature generation stage and the signature verification stage. The signature generation stage includes one ECSM (Q=kP), where both *P* and *Q* are two points over a given elliptic curve, and *k* is a scalar. The signature verification stage requires an ECDSM. ECDSM (kP+lQ) consists of two ECSMs and a point addition (PA). The three-level hierarchy of ECC cryptosystems is illustrated in Figure A1. Both ECSM and ECDSM can be computed by calculating PA and point doubling (PD) iteratively. PA ($P_{PA}=P_{1}+P_{2}$) and PD ($P_{PD}=2P_{1}$) are defined geometrically by the chord-and-tangent rule over finite fields.

Due to the carry-free property, the arithmetic over binary finite fields $GF(2^{m})$ is more suitable for hardware implementation. Over $GF(2^{m})$, a non-supersingular elliptic curve E is defined as shown in Equation (1) with parameters *a* and *b*.

(A2) $$E:y^{2}+xy=x^{3}+ax^{2}+b.$$

Given $P_{1}=\left(x_{1},y_{1}\right)$ and $P_{2}=\left(x_{2},y_{2}\right)$, PA and PD are as follows:

(A3) $$P_{PA}\left(x_{PA},y_{PA}\right)=P_{1}+P_{2}=\left\{\begin{array}{c} x_{PA}=\lambda^{2}+\lambda +x_{1}+x_{2}+a\\ y_{PA}=\lambda \left(x_{1}+x_{PA}\right)+x_{PA}+a \end{array}\right.$$

(A4) $$P_{PD}\left(x_{PD},y_{PD}\right)=2P_{1}=\left\{\begin{array}{c} x_{PD}=\gamma^{2}+\gamma +a=x_{1}^{2}+\frac{b}{x_{1}^{2}}\\ y_{PD}=x_{1}^{2}+\gamma x_{PD}+x_{PD} \end{array}\right.$$

where $\lambda =\frac{y_{1}+y_{2}}{x_{1}+x_{2}}$ and $\gamma =x_{1}+\frac{y_{1}}{x_{1}}$.

Note that the result of the *x*-coordinate in PD can be further simplified as $x_{1}^{2}+\frac{b}{x_{1}^{2}}$, which is an intrinsic property of the elliptic curve over $GF(2^{m})$. If we introduce the differential addition chain (DAC) to guarantee the relation $P_{2}=P_{1}+P$ in the iteration, then the expression of PA can be further simplified as $x_{3}=x+{\left(\frac{x_{1}}{x_{1}+x_{2}}\right)}^{2}+\frac{x_{1}}{x_{1}+x_{2}}$, and the derivation is shown below.

From Equation (2), we have

(A5) $$x_{PA}=\frac{x_{1}y_{2}+x_{2}y_{1}+x_{1}x_{2}^{2}+x_{1}^{2}x_{2}}{x_{1}^{2}+x_{2}^{2}}$$

by simplifying the numerator with $2b=2y^{2}+2xy+2x^{3}+2ax^{2}$, since the items with even coefficients can be eliminated over $GF(2^{m})$. With the relation $P=P_{2}-P_{1}$, we can obtain $-P_{1}=\left(x_{1},x_{1}+y_{1}\right)$. Thus, we have another similar relation:

(A6) $$x=\frac{x_{1}y_{2}+x_{2}\left(x_{1}+y_{1}\right)+x_{1}x_{2}^{2}+x_{1}^{2}x_{2}}{x_{1}^{2}+x_{2}^{2}}.$$

Comparing Equation (4) and Equation (5), we also simplify the expression of PA as

(A7) $$x_{3}=x+\frac{x_{1}x_{2}}{{\left(x_{1}+x_{2}\right)}^{2}}=x+\frac{2x_{1}^{2}+x_{1}x_{2}}{{\left(x_{1}+x_{2}\right)}^{2}}=x+{\left(\frac{x_{1}}{x_{1}+x_{2}}\right)}^{2}+\frac{x_{1}}{x_{1}+x_{2}}.$$

Considering simplified expressions for PD and PA not involving *y*-coordinates, the calculation of *y*-coordinates during the iteration of PA and PD in ECSM can be eliminated, as shown below.

(A8) $$\left\{\begin{array}{c} PD:\;x_{PD}=x_{1}^{2}+\frac{b}{x_{1}^{2}}\\ PA:\;x_{PA}=x+{\left(\frac{x_{1}}{x_{1}+x_{2}}\right)}^{2}+\frac{x_{1}}{x_{1}+x_{2}} \end{array}\right.for\;P_{2}=P+P_{1}$$

Executing PA and PD in affine coordinates requires multiple inversions during the iteration. Inversions over $GF(2^{m})$ are time-consuming. Therefore, we can map the points from affine coordinates (x,y) to Lopez–Dahab (LD) projective coordinates (X,Y,Z). As a result, we only need to calculate multiplications, additions, and squares in each iteration, leaving only one inversion at the end of ECSM. The expressions of PA and PD in LD projective coordinates are:

(A9) $$\left\{\begin{array}{c} X_{PA}=x_{P}{\left(X_{1}Z_{2}+X_{2}Z_{1}\right)}^{2}+X_{1}X_{2}\cdot Z_{1}\cdot Z_{2}\\ Z_{PA}={\left(X_{1}Z_{2}+X_{2}Z_{1}\right)}^{2} \end{array}\right.$$

(A10) $$\left\{\begin{array}{c} X_{PD}={X_{1}}^{4}+b{Z_{1}}^{4}\\ Z_{PD}=X_{1}^{2}{Z_{1}}^{2} \end{array}\right.$$

In the end, the recovery of *y* and coordinate conversion is presented as:

(A11) $$\left\{\begin{array}{c} x_{Q}=\frac{X_{1}}{Z_{1}}\\ y_{Q}=y_{P}+\frac{\left(x_{P}+x_{Q}\right)\left[\left(X_{1}+x_{P}Z_{1}\right)\left(X_{2}+x_{P}Z_{2}\right)+\left(x_{P}^{2}+y_{P}\right)Z_{1}Z_{2}\right]}{x_{P}Z_{1}Z_{2}}. \end{array}\right.$$

With the simplified PA and PD expressions in Equations (A9) and (A10), the Montgomery-ladder-based ECSM algorithm is given in Algorithm A4. Note that the recovery of *y* in the Montgomery-ladder-based ECSM algorithm adopts Equation (A11).

**Algorithm A4** The Montgomery Ladder ECSM Algorithm
**Require:** $k={\left(k_{t-1},\cdots ,k_{1},k_{0}\right)}_{2}$ with $k_{t-1}=1$, $P=(x_{P},y_{P})$. **Ensure:** Q=kP. 1: Set $P_{1}\leftarrow \left(x_{P},1\right),P_{2}\leftarrow \left(x_{P}^{4}+b,x_{P}^{2}\right)$. 2: **for** i=t−2 to 0 **do** 3: **if** $k_{i}=1$ **then** 4: $P_{1}\leftarrow PA\left(P_{1},P_{2}\right)$, 5: $P_{2}\leftarrow PD\left(P_{2}\right)$, 6: **else** 7: $P_{2}\leftarrow PA\left(P_{1},P_{2}\right)$, 8: $P_{1}\leftarrow PD\left(P_{1}\right)$, 9: **end if** 10: **end for** 11: Recover *y* in affine coordinates. **Return *Q***.

Meanwhile, if only one single PA is executed, then we should adopt the single PA algorithm in LD coordinates as shown in Algorithm A5 rather than Equation (A9).

**Algorithm A5** The Single PA Algorithm
**Require: $P_{1}=\left(X_{1},Y_{1},Z_{1}\right)$ and $P_{2}=\left(X_{2},Y_{2},Z_{2}\right)$**. **Ensure: $P_{3}=\left(X_{3},Z_{3}\right)$ and $x_{p}$**. 1: $X_{1}\leftarrow X_{1}Z_{2},X_{2}\leftarrow X_{2}Z_{1}$ 2: $Y_{1}\leftarrow Y_{1}Z_{2},X_{2}\leftarrow Y_{2}Z_{1}$ 3: $X_{1}\leftarrow X_{1}+X_{2}$ 4: $Y_{1}\leftarrow Y_{1}+Y_{2}$ 5: $Y_{1}\leftarrow Y_{1}Y_{2},Z_{1}\leftarrow Z_{1}Z_{2},X_{2}\leftarrow X_{1}^{2}$ 6: $Y_{1}\leftarrow Y_{1}Z_{1},Z_{3}\leftarrow Z_{1}X_{2},X_{1}\leftarrow X_{1}+Z_{1}$ 7: $X_{1}\leftarrow X_{1}X_{2}$ 8: $X_{3}\leftarrow Y_{1}+X_{1}$ 9: $x_{p}\leftarrow X_{3}/Z_{3}$ **Return: $X_{3},Z_{3},x_{p}$**.

## Appendix C. Differential Addition Chain

In a DAC, each sum in the chain has already been accompanied by a difference: i.e., whenever a new element $C_{new}=C_{1}+C_{2}$ is formed by adding two existing elements $C_{1}$ and $C_{2}$, the difference of two elements $C_{1}-C_{2}$ was already in this chain. We can efficiently calculate ECDSM with a two-dimensional DAC, as shown in Figure A2. The left side of Figure A2 lists all elements in the two-dimensional DAC, and the right side provides flags for each loop. We only need to precompute the flag signal for each round before initiating the main loop of PA and PD iterations. These flag signals are used to determine the two-dimensional DAC. Subsequently, the main loop is initiated and is entirely controlled based on these flag signals to execute PA and PD. Each round of iteration involves two PAs and one PD. Therefore, the iteration process is completely uniform, making it resilient against some power analysis attacks. Moreover, since the differential relationships always hold throughout each round of iteration, the simplified forms in Equations (A9) and (A10) can consistently be utilized.

## Appendix D. Custom ECDSA Signature and Verification

**Algorithm A6** Custom ECDSA Signature cusECDSA_SIG
**Require: *d*, *m* and *n*.** **Ensure: $x_{p}$ and $s^{-1}\;mod\;n$.** 1: **while** $s^{-1}\;mod\;n\neq 0$ **do** 2: **while** $x_{p}\neq 0$ **do** 3: Select *k* from 1 to n−1 randomly; 4: $P=kG=(x_{p},y_{p})$; 5: **end while** 6: Z=HASH(m); 7: $s^{-1}\;mod\;n=k{\left(z+rd\right)}^{-1}\;mod\;n$; 8: **end while** **Return: $x_{p}$ and $s^{-1}\;mod\;n$.**

**Algorithm A7** Custom ECDSA Verification cusECDSA_VER
**Require: $x_{p}$, $x_{p}z\;mod\;p$, $s^{-1}\;mod\;n$, *m*, *H*.** **Ensure: Verification results, *X*, *Z*.** 1: z=HASH(m); 2: $u_{1}=s^{-1}z\;mod\;n$; 3: $u_{2}=s^{-1}x_{p}\;mod\;n$; 4: $P=u_{1}G+u_{2}H=\left(X,Z\right)$; 5: **if** $X=x_{p}Z$ **then** 6: The verification passes. 7: **else** 8: The verification fails. 9: **end if** **Return: *X* and *Z*.**

## References

[1] Arora H., Soni G.K., Kushwaha R.K., Prasoon P. (2021). Digital image security based on the hybrid model of image hiding and encryption. Proceedings of the 2021 6th International Conference on Communication and Electronics Systems (ICCES).

[2] Matted S., Shankar G., Jain B.B. (2021). Enhanced image security using stenography and cryptography. Computer Networks and Inventive Communication Technologies.

[3] Halak B., Zwolinski M., Mispan M.S. (2016). Overview of PUF-based hardware security solutions for the Internet of Things. Proceedings of the 2016 IEEE 59th International Midwest Symposium on Circuits and Systems (MWSCAS).

[4] Mall P., Amin R., Das A.K., Leung M.T., Choo K.K.R. (2022). PUF-based authentication and key agreement protocols for IoT, WSNs and smart grids: A comprehensive survey. IEEE Internet Things J..

[5] Holcomb D.E., Burleson W.P., Fu K. (2008). Power-up SRAM state as an identifying fingerprint and source of true random numbers. IEEE Trans. Comput..

[6] van Dijk M., Rührmair U. (2014). Protocol attacks on advanced PUF protocols and countermeasures. Proceedings of the 2014 Design, Automation & Test in Europe Conference & Exhibition (DATE).

[7] Rührmair U., van Dijk M. (2013). PUFs in security protocols: Attack models and security evaluations. Proceedings of the 2013 IEEE Symposium on Security and Privacy.

[8] Rührmair U., Jaeger C., Algasinger M. (2011). An attack on PUF-based session key exchange and a hardware-based countermeasure: Erasable PUFs. Proceedings of the International Conference on Financial Cryptography and Data Security.

[9] Karakoyunlu D., Sunar B. (2010). Differential template attacks on PUF enabled cryptographic devices. Proceedings of the 2010 IEEE International Workshop on Information Forensics and Security.

[10] Merli D., Schuster D., Stumpf F., Sigl G. (2011). Side-channel analysis of PUFs and fuzzy extractors. Proceedings of the International Conference on Trust and Trustworthy Computing.

[11] Patterson D.A., Hennessy J.L. (2016). Computer Organization and Design ARM Edition: The Hardware Software Interface.

[12] Lohrke H., Tajik S., Krachenfels T., Boit C., Seifert J.P. (2018). Key extraction using thermal laser stimulation: A case study on xilinx ultrascale fpgas. IACR Trans. Cryptogr. Hardw. Embed. Syst..

[13] Hankerson D., Menezes A.J., Vanstone S. (2006). Guide to Elliptic Curve Cryptography.

[14] Rashid M., Imran M., Jafri A.R., Al-Somani T.F. (2019). Flexible architectures for cryptographic algorithms—A systematic literature review. J. Circuits Syst. Comput..

[15] Li J., Li Z., Cao S., Zhang J., Wang W. (2019). Speed-Oriented Architecture for Binary Field Point Multiplication on Elliptic Curves. IEEE Access.

[16] Khan Z.U.A., Benaissa M. (2015). Throughput/Area-efficient ECC Processor Using Montgomery Point Multiplication on FPGA. IEEE Trans. Circuits Syst. II Express Briefs.

[17] Khan Z.U.A., Benaissa M. (2017). High-Speed and Low-Latency ECC Processor Implementation Over GF( 2^m^) on FPGA. IEEE Trans. Very Large Scale Integr. VLSI Syst..

[18] Imran M., Rashid M., Jafri A.R., Najam-Ul-Islam M. (2018). ACryp-Proc: Flexible asymmetric crypto processor for point multiplication. IEEE Access.

[19] Harb S., Jarrah M. (2019). FPGA implementation of the ECC over GF (2 m) for small embedded applications. ACM Trans. Embed. Comput. Syst. TECS.

[20] Kiyan T., Lohrke H., Boit C. (2018). Comparative assessment of optical techniques for semi-invasive SRAM data read-out on an MSP430 microcontroller. Proceedings of the ISTFA 2018: Proceedings from the 44th International Symposium for Testing and Failure Analysis.

[21] Faraj M., Gebotys C. (2021). Quiescent photonics side channel analysis: Low cost SRAM readout attack. Cryptogr. Commun..

[22] Shifman Y., Miller A., Keren O., Weizmann Y., Shor J. (2018). A Method to Improve Reliability in a 65-nm SRAM PUF Array. IEEE Solid-State Circuits Lett..

[23] Satpathy S., Mathew S.K., Suresh V., Anders M.A., Kaul H., Agarwal A., Hsu S.K., Chen G., Krishnamurthy R.K., De V.K. (2017). A 4-fJ/b delay-hardened physically unclonable function circuit with selective bit destabilization in 14-nm trigate CMOS. IEEE J.-Solid-State Circuits.

[24] Alvarez A., Zhao W., Alioto M. (2015). 14.3 15fJ/b static physically unclonable functions for secure chip identification with <2% native bit instability and 140× Inter/Intra PUF hamming distance separation in 65 nm. Proceedings of the 2015 IEEE International Solid-State Circuits Conference-(ISSCC) Digest of Technical Papers.

[25] Yang K., Dong Q., Blaauw D., Sylvester D. (2017). 8.3 A 553F 2 2-transistor amplifier-based Physically Unclonable Function (PUF) with 1.67% native instability. Proceedings of the 2017 IEEE International Solid-State Circuits Conference (ISSCC).

[26] Yamamoto D., Sakiyama K., Iwamoto M., Ohta K., Takenaka M., Itoh K. (2013). Variety enhancement of PUF responses using the locations of random outputting RS latches. J. Cryptogr. Eng..

[27] Zhang F., Yang S., Plusquellic J., Bhunia S. (2016). Current based PUF exploiting random variations in SRAM cells. Proceedings of the 2016 Design, Automation & Test in Europe Conference & Exhibition (DATE).

[28] Kumar S.S., Guajardo J., Maes R., Schrijen G.J., Tuyls P. (2008). The butterfly PUF protecting IP on every FPGA. Proceedings of the 2008 IEEE International Workshop on Hardware-Oriented Security and Trust.

[29] Bossuet L., Ngo X.T., Cherif Z., Fischer V. (2013). A PUF based on a transient effect ring oscillator and insensitive to locking phenomenon. IEEE Trans. Emerg. Top. Comput..

[30] Itoh T., Tsujii S. (1988). A fast algorithm for computing multiplicative inverses in GF (2m) using normal bases. Inf. Comput..

[31] Khan Z.U.A., Benaissa M. High speed ECC implementation on FPGA over GF(2^m^). Proceedings of the International Conference on Field Programmable Logic and Applications.

[32] Imran M., Rashid M., Jafri A.R., Kashif M. (2019). Throughput/area optimised pipelined architecture for elliptic curve crypto processor. IET Comput. Digit. Tech..

[33] Nguyen T.T., Lee H. (2016). Efficient algorithm and architecture for elliptic curve cryptographic processor. J. Semicond. Technol. Sci..

[34] Imran M., Shafi I., Jafri A.R., Rashid M. (2017). Hardware design and implementation of ECC based crypto processor for low-area-applications on FPGA. Proceedings of the 2017 International Conference on Open Source Systems & Technologies (ICOSST).

